# Ubiquitin initiates sorting of Golgi and plasma membrane proteins into the vacuolar degradation pathway

**DOI:** 10.1186/1471-2229-12-164

**Published:** 2012-09-12

**Authors:** David Scheuring, Fabian Künzl, Corrado Viotti, Melody San Wan Yan, Liwen Jiang, Swen Schellmann, David G Robinson, Peter Pimpl

**Affiliations:** 1 Department of Developmental Genetics, Center for Plant Molecular Biology (ZMBP), University of Tübingen, Tübingen, 72076, Germany; 2 Department of Plant Cell Biology, Centre for Organismal Studies, University of Heidelberg, Heidelberg, 69120, Germany; 3 Plant Developmental Biology, Centre for Organismal Studies, University of Heidelberg, Heidelberg, 69120, Germany; 4 School of Life Sciences, Centre for Cell and Developmental Biology, The Chinese University of Hong Kong, Shatin NT, Hong Kong, PR China; 5 Botanical Institute, Biozentrum Köln, University of Cologne, Cologne, 50674, Germany

## Abstract

**Background:**

In yeast and mammals, many plasma membrane (PM) proteins destined for degradation are tagged with ubiquitin. These ubiquitinated proteins are internalized into clathrin-coated vesicles and are transported to early endosomal compartments. There, ubiquitinated proteins are sorted by the endosomal sorting complex required for transport (ESCRT) machinery into the intraluminal vesicles of multivesicular endosomes. Degradation of these proteins occurs after endosomes fuse with lysosomes/lytic vacuoles to release their content into the lumen. In plants, some PM proteins, which cycle between the PM and endosomal compartments, have been found to be ubiquitinated, but it is unclear whether ubiquitin is sufficient to mediate internalization and thus acts as a primary sorting signal for the endocytic pathway. To test whether plants use ubiquitin as a signal for the degradation of membrane proteins, we have translationally fused ubiquitin to different fluorescent reporters for the plasma membrane and analyzed their transport.

**Results:**

Ubiquitin-tagged PM reporters localized to endosomes and to the lumen of the lytic vacuole in tobacco mesophyll protoplasts and in tobacco epidermal cells. The internalization of these reporters was significantly reduced if clathrin-mediated endocytosis was inhibited by the coexpression of a mutant of the clathrin heavy chain, the clathrin hub. Surprisingly, a ubiquitin-tagged reporter for the Golgi was also transported into the lumen of the vacuole. Vacuolar delivery of the reporters was abolished upon inhibition of the ESCRT machinery, indicating that the vacuolar delivery of these reporters occurs via the endocytic transport route.

**Conclusions:**

Ubiquitin acts as a sorting signal at different compartments in the endomembrane system to target membrane proteins into the vacuolar degradation pathway: If displayed at the PM, ubiquitin triggers internalization of PM reporters into the endocytic transport route, but it also mediates vacuolar delivery if displayed at the Golgi. In both cases, ubiquitin-tagged proteins travel via early endosomes and multivesicular bodies to the lytic vacuole. This suggests that vacuolar degradation of ubiquitinated proteins is not restricted to PM proteins but might also facilitate the turnover of membrane proteins in the early secretory pathway.

## Background

The endocytic uptake of proteins and lipids is the driving force that establishes and maintains cellular polarity, but also allows for intercellular communication and facilitates interactions with the environment [1,2]. Endocytosis involves invagination and fission of vesicles at the plasma membrane (PM) and their transport to endosomes. Endocytosis in walled plant cells has been shown to exist by the use of fluorescent dyes in the early 2000s and has been confirmed by the subsequent identification of endocytic cargo molecules like the auxin efflux facilitator PINFORMED 1 (PIN1) [3] or cell surface receptors like the brassinosteroid receptor BRASSINOSTEROID INSENSITIVE 1 (BRI1) and the flagellin receptor FLAGELLIN-SENSING 2 (FLS2) [4-6].

In yeast and mammals, the uptake of certain membrane proteins from the PM requires ubiquitin as an internalization signal [7-9]. Ubiquitin is a highly conserved protein that is found in all eukaryotes ranging from unicellular organisms to mammals and higher plants [10]. Ubiquitination is one of the most common *post*-translational protein modifications being responsible for proteasomal degradation, membrane transport events, DNA repair and other mechanisms such as signaling and cell cycle control [11]. The C-terminus of ubiquitin is able to form covalent bonds with other proteins and once a single ubiquitin moiety is bound, it can be conjugated with additional ubiquitin molecules in a process called poly-ubiquitination [12,13]. Here, number and spatial orientation of added ubiquitin entities are crucial for a protein’s destiny [8,14,15]. In this context, poly-ubiquitination of soluble proteins results in their cytosolic degradation by the 26S proteasome [16,17], while the attachment of a single ubiquitin monomer to membrane-bound proteins facilitates sorting into intralumenal vesicles (ILVs) of late endosomes (LEs/MVBs, multivesicular bodies) followed by lysosomal degradation [18]. However, details about the number of required ubiquitin moieties to trigger internalization at the PM are still controversially discussed [19].

In plants, FLS2 internalization at the PM is triggered by flg22, a 22 amino acid epitope of bacterial flagellin. In the presence of this elicitor, FLS2 was found to be ubiquitinated [20], but it is unclear whether this ubiquitination represents the sorting signal for its endocytic uptake. It has been shown that down-regulation of the PM-localized iron transporter IRON-REGULATED TRANSPORTER 1 (IRT1) involves multiple mono-ubiquitinations [21]. In the case of BORON TRANSPORTER 1 (BOR1), however, down-regulation requires the combined action of ubiquitin and tyrosine-based sorting signals for internalization and for endosomal sorting [22]. Very recently, ubiquitination of PIN2 was shown to be essential for its function in root gravitropism [23] and translational fusion of ubiquitin to PIN2 or to the plasma membrane ATPase PMA, which mimics constitutive ubiquitination, was shown to alter the localization and stability of these proteins [23,24]. These findings suggest that ubiquitin-dependent sorting mechanisms for PM proteins also exist in plants. However, vacuolar degradation of some integral PM proteins does not necessarily depend on ubiquitination as has been reported for the RICE SECRETORY CARRIER MEMBRANE PROTEIN 1 (OsSCAMP1) and the leucine-rich repeat receptor-like kinase AtLRR84A [25].

A common step in the vacuolar degradation pathway of membrane proteins is their sorting into ILVs of endosomes. This process is mediated by four ESCRT (endosomal sorting complex required for transport) complexes, termed ESCRT-0 to ESCRT-III [18]. ESCRT-0 but also ESCRT-I and ESCRT-II recognize and concentrate ubiquitinated cargo and recruit ESCRT-III. This complex recruits in turn ESCRT-associated proteins like the deubiquitinating enzyme Doa4/UBPY and the AAA-ATPase Vps4/SKD1 and drives the formation of the intralumenal vesicles, resulting in the formation of MVBs [18]. ESCRT homologues have been shown to exist in plants [26-31] although molecular interactions between ubiquitinated cargo and ESCRT components have not yet been demonstrated. Nonetheless, the cytokinesis-specific syntaxin KNOLLE as well as PIN1, BRI1 and the vacuolar sorting receptor BP80 all locate to the ILVs of MVBs [32-35]. Moreover, the localization of PIN1, PIN2 and the auxin influx carrier AUX1 was found to be dependent on the function of the ESCRT machinery [34]. Together, this suggests that ESCRT-mediated sorting contributes to the regulation of membrane proteins via vacuolar degradation.

However, degradation does not necessarily have to follow endocytosis, since PIN1 and BRI1 also cycle constitutively between the PM and endosomes [3,36]. The signals that mediate protein sorting into the endocytic, the recycling or the degradation pathways in plants are not yet fully understood. The analysis of sorting determinants for individual transport steps within this complex network of transport routes is further complicated by the fact that PM proteins reach the PM via the secretory pathway which merges with the endocytic route at the *trans*-Golgi network (TGN)/early endosome (EE) [35,37,38]. Hence, it is difficult to judge whether a given protein that localizes to this compartment has just been internalized or is still on its way to its primary destination. To overcome this problem and to analyze specific sorting signals for individual transport routes, we have prepared conceptually different fluorescent PM reporters. The first class of reporters is *post*-translationally inserted into the PM, which determines the internalization at the PM as the first possible transport step. These reporters are based on the observation that the 26 C-terminal residues of the *Arabidopsis* type-II ROP-GTPase AtROP10 are sufficient to cause PM attachment when fused to the C-terminus of cytosolic GFP [39]. The respective sequence contains a 15 amino acid polybasic domain followed by a highly conserved motif. This motif consists of two glycine/cysteine pairs flanking 5–6 non-specified residues and is known as the [GC-CG] box. The *post*-translational PM recruitment is supposed to occur after S-acylation of the two cysteines [39]. The second class of reporters is based on type-I transmembrane proteins, which are delivered to the PM via the secretory pathway.

Here, we show, that ubiquitin is sufficient to target the *post*-translationally inserted PM reporter Box-GFP-Ub and the transmembrane protein reporter RFP-TMD23-Ub into the endocytic pathway. Interestingly, ubiquitin was also found to be sufficient to target the Golgi-localized transmembrane protein RFP-TMD20 into the lumen of the lytic vacuole. The vacuolar delivery of these reporters can be inhibited when a mutagenized ESCRT-associated component (AtSKD1(AQ)) is expressed. The use of reporters carrying a mutagenized derivative of ubiquitin furthermore reveals different ubiquitin requirements for the internalization at the PM compared to the ubiquitin-mediated sorting at the Golgi. Together, these results show that ubiquitin acts as a signal for vacuolar degradation of membrane proteins and is not restricted to sorting events at the PM.

## Results

### Ubiquitin causes internalization of a non-secretory reporter at the PM

**Figure 1:**
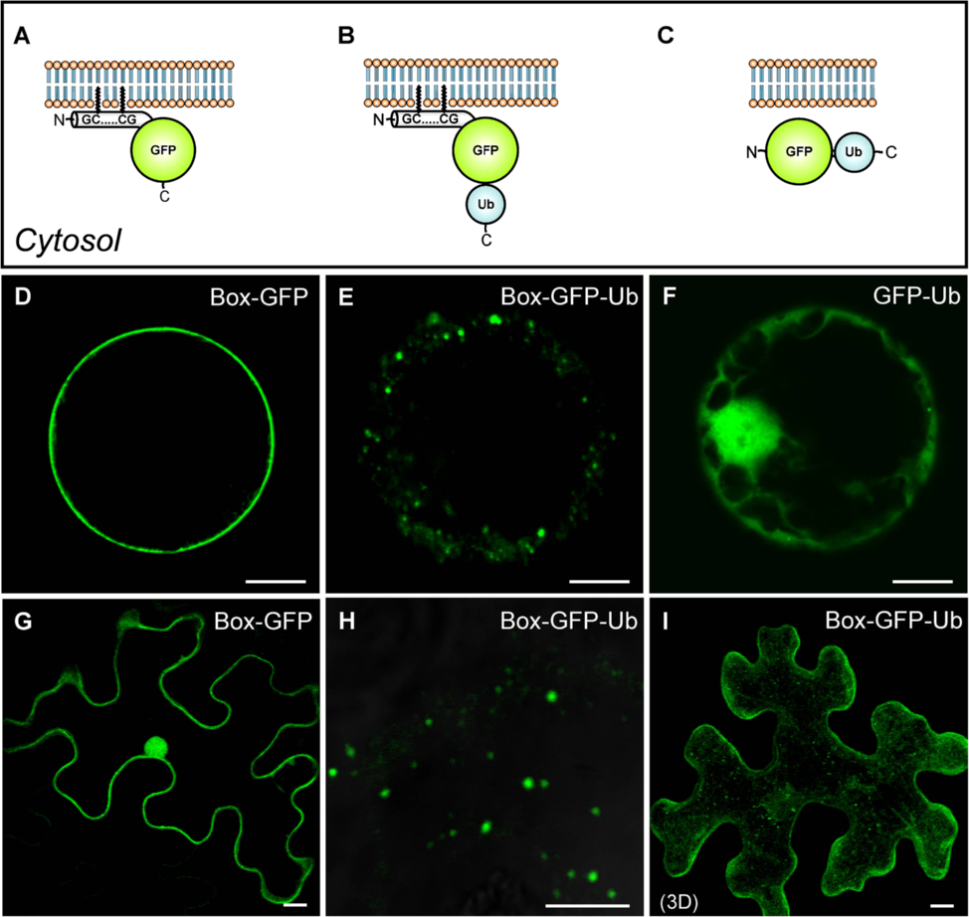
**Expression of non-secretory reporters to analyze the endocytic pathway.A-C**: schematic representation of the constructs Box-GFP (**A**), Box-GFP-Ub (**B**) and GFP-Ub (**C**). **D-F**: tobacco mesophyll protoplasts were transfected with the corresponding plasmids as indicated below. (**D**) Box-GFP is efficiently recruited onto the PM, while Box-GFP-Ub (**E**) predominantly localizes to punctae. **F**: GFP-Ub is homogenously distributed throughout the cytoplasm and the nuclear matrix. **G-I**: Localization of the reporters given above *in planta*, confirming the results from (**D-E**). **I**: 3D projection of a leaf epidermal cell expressing Box-GFP-Ub. Scale bars = 5 μm.

**Figure 2:**
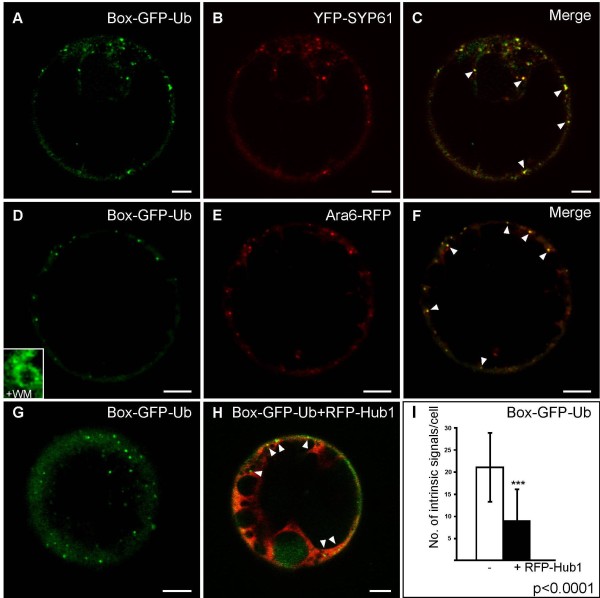
**Ubiquitin-dependent internalization of Box-GFP-Ub.** Expression of markers/reporters in tobacco mesophyll protoplasts as indicated below. **A-C**: Box-GFP-Ub (green) and the TGN/EE marker YFP-SYP61 (red) partially colocalize (arrowheads). **D-F**: Box-GFP-Ub (green) and MVB/LE marker Ara6-RFP (red) colocalize to a high degree (arrowheads). Box-GFP-Ub localizes to characteristic ring-like MVB structures after wortmannin-treatment (+WM; inset in **D**). For quantitative analysis of colocalization see Additional file 2. Endosomal signals of Box-GFP-Ub (**G**) are significantly reduced in quantity if coexpressed with the clathrin hub (RFP-Hub1; red, **H**). Arrowheads indicate remaining Box-GFP-Ub-positive endosomes. **I**: Statistical analysis reveals that the punctate signals are about 57% less abundant in cells coexpressing the RFP-Hub1 (+) as compared to control cells (−). (***) p <0.0001. Scale bars = 5 μm.

To analyze sorting signals for the endocytic pathway, we have created GFP-based reporters that are *post*-translationally inserted into the PM but also allow the analysis of internalization signals by fusing the 26 C-terminal residues of AtROP10 (hereafter named Box) to the N-terminus of GFP (Figure 1A). To test whether ubiquitin causes internalization of proteins from the PM, we fused ubiquitin (Ub) to the C-terminus of Box-GFP, resulting in the construct Box-GFP-Ub (Figure 1B). We also generated a control construct lacking the N-terminal Box sequence (GFP-Ub), to assess the requirement for PM insertion (Figure 1C). Expression of these reporters in tobacco mesophyll protoplasts shows that Box-GFP is localized to the PM (Figure 1D), indicating efficient recruitment from the cytosol to the PM. It also demonstrates that the function of the Box is independent of its C- or N-terminal position at the reporter. In sharp contrast, the vast majority of Box-GFP-Ub localized to discrete punctae (Figure 1E, Additional file 1), indicating successful internalization of the reporter. Neither discrete punctae nor PM signals were observed in case of GFP-Ub (Figure 1F). As expected for this cytosolic reporter, fluorescence was distributed throughout the cytoplasm and the nuclear matrix. This suggests that the punctae of the reporter Box-GFP-Ub depend on membrane association and the presence of ubiquitin. To rule out that the observed localization might be due to specific properties of protoplasts, we have expressed the reporters in tobacco leaves via *Agrobacterium*-mediated transfection. The expression of Box-GFP in epidermal cells revealed its localization at the PM (Figure 1G), while Box-GFP-Ub was again found to label numerous punctate structures (Figure 1H and I). This shows that the localization of the reporters in protoplasts and *in planta* is the same. To analyze whether the punctate Box-GFP-Ub structures represent endosomes, we performed co-expression experiments with markers for the TGN/EE (YFP-SYP61; [40,41]), the MVB/LE (Ara6-RFP; [40]) and the Golgi (Man1-RFP; [42]). In these experiments, Box-GFP-Ub partially colocalized with YFP-SYP61 (Figure 2A-C, Additional file 2A) and Ara6-RFP (Figure 2D-F, Additional file 2B). LE/MVB-localization of Box-GFP-Ub is furthermore supported by the appearance of the fluorescent signals in ring-like structures after incubation with the drug wortmannin (WM, inset in Figure 2D), which form as a result of the drug-induced fusion of LEs/MVBs [43]. Box-GFP-Ub signals did not overlap with the signals of the Golgi marker (Additional file 2C), suggesting that the localization of Box-GFP-Ub is restricted to endosomes.

We next analyzed whether Box-GFP-Ub reaches its endosomal localization via the endocytic pathway, a transport that involves the formation of clathrin-coated vesicles at the PM. It was shown that this process is inhibited by the expression of a truncated mutant of the clathrin heavy chain, the clathrin hub [28,44-46]. Therefore we compared the numbers of punctate Box-GFP-Ub signals in control cells, expressing only Box-GFP-Ub (Figure 2G), with signals in cells coexpressing the clathrin hub (RFP-Hub1, Figure 2H). Figure 2I shows that the number of Box-GFP-Ub signals is significantly reduced upon RFP-Hub1 coexpression (Figure 2H-I), confirming that the endosomal localization of Box-GFP-Ub is indeed due to internalization at the PM via clathrin-mediated endocytosis.

### Ubiquitin causes a plasma membrane protein to traffic to the vacuole

The previous experiments showed that a PM-associated protein can be efficiently internalized by a C-terminal fusion to ubiquitin. However, the fluorescent reporter was transported along the endocytic route only as far as the MVB/LE but could not be detected in the vacuole, even when expressed for 48 hours (data not shown). Since fluorescent signals of GFP-based reporters can be routinely detected in the lytic vacuole under these experimental conditions (Additional file 3, compare A and B), we conclude that the lack of vacuolar fluorescence of the Box-GFP-Ub is not due to vacuolar degradation, but instead due to a failure of vacuolar delivery. One reason for this could be that the membrane association via S-acylation is not stable enough to survive MVB-mediated sorting. Alternatively, the translational fusion of ubiquitin to the reporter prevents deubiquitination, a requirement for vacuolar delivery in yeast [47].

**Figure 3:**
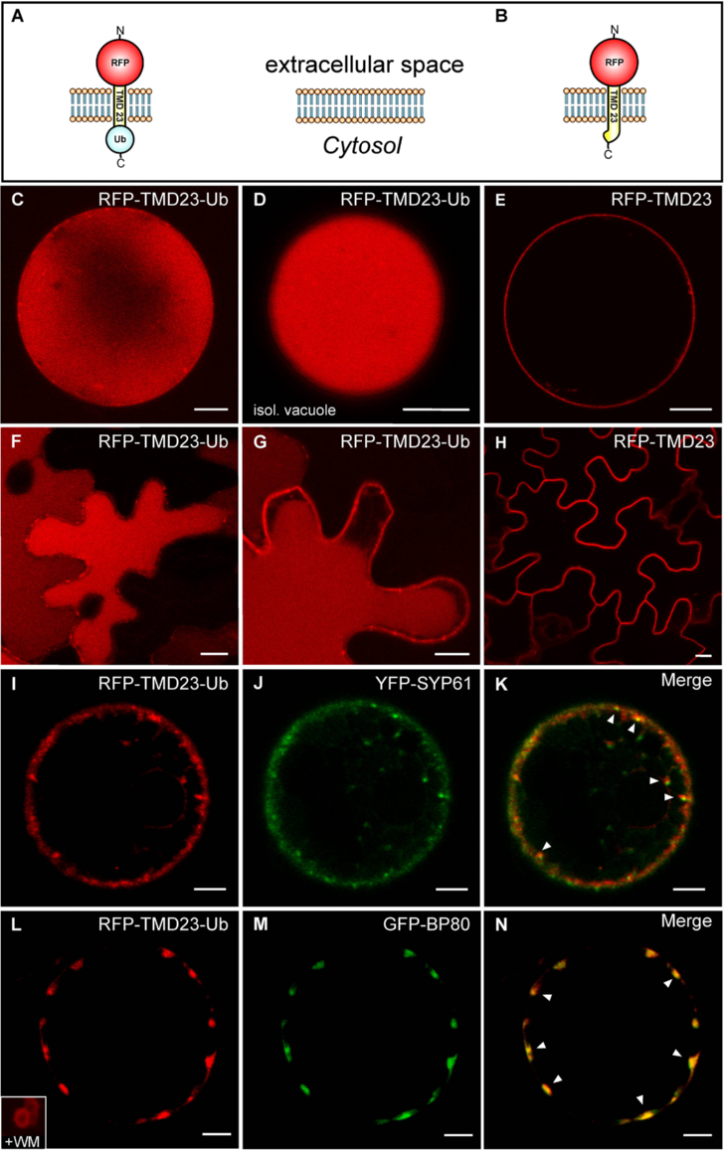
**Ubiquitin-dependent transport of an integral PM protein to the vacuole.A-B**: Schematic representation of the constructs RFP-TMD23-Ub (**A**) and RFP-TMD23 (**B**). Localization of the constructs in tobacco mesophyll protoplasts after 36 h (C-E, to allow for vacuolar accumulation) or 24 h (**I-N**, for colocalization analysis) and leaf epidermal cells (**F-H**). **C**: RFP-TMD23-Ub is targeted into the vacuolar lumen. (**D**) Isolated vacuole. (**E**) RFP-TMD23 localizes to the PM. F-H: Localization of the reporters given above *in planta*, confirming the results from **C-E. I-K**: RFP-TMD23-Ub (red) and YFP-SYP61 (green) partially colocalize (arrowheads). **L-N**: RFP-TMD23-Ub (red) and MVB/LE marker GFP-BP80 (green) overlap in punctate structures (arrowheads). RFP-TMD23-Ub localizes to ring-like MVB structures after wortmannin-treatment (+WM; inset in **L**). Scale bars = 5 μm. For quantitative analysis of colocalization see Additional file 2.

To answer this question, we have prepared a PM-targeted ubiquitin fusion construct based on a type-I transmembrane protein (RFP-TMD23-Ub, Figure 3A) by adding the ubiquitin sequence to the C-terminus of the PM marker RFP-TMD23, (Figure 3B; TM23 in [48]). In contrast to the lipid-anchored Box-GFP-Ub (see Figure 1B, E, H, I), expression of RFP-TMD23-Ub yields a vacuolar pattern in protoplasts and isolated vacuoles (Figure 3C, D), which was never observed for RFP-TMD23, lacking a C-terminal ubiquitin (Figure 3e). Comparison of RFP-TMD23-Ub with the vacuolar reporter spL-RFP [49] furthermore reveals that both molecules are equally well delivered to the vacuole (Additional file 3C, D). The same vacuolar pattern can be seen when RFP-TMD23-Ub is analyzed *in planta* (Figure 3F-H), indicating that sorting and transport mechanisms of these reporters do not differ between both experimental systems. Together, this suggests that it is the type of membrane association rather than the requirement for deubiquitination that prevented the vacuolar delivery of the Box-GFP-Ub reporter.

The RFP-TMD23-Ub signal was not restricted to the vacuolar lumen but was also detected at the PM and was occasionally associated with organelles in the peripheral cytoplasm. We therefore performed coexpression experiments with markers for TGN/EE (YFP-SYP61) and MVB/LE (GFP-BP80; [50]). This analysis was performed with detection parameters that reduced the strong and diffuse vacuolar background of RFP-TMD23-Ub. A large number of the punctate signals overlapped with the markers for the TGN/EE (Figure 3I-K, additional file D) and the MVB/LE (Figure 3L-N, inset in 3 L and additional file E), both of which are transit compartments along the vacuolar route.

**Figure 4:**
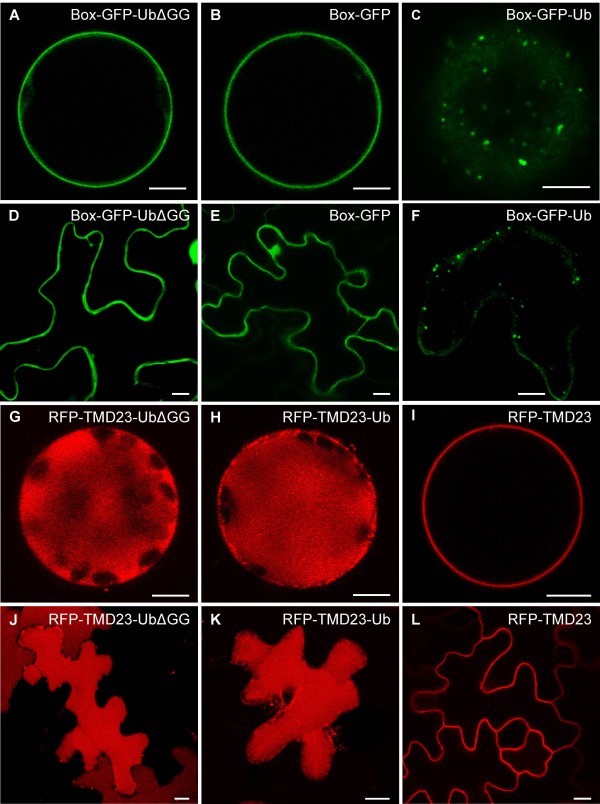
**The C-terminus of ubiquitin is important for PM internalization.** Tobacco mesophyll protoplasts and leaf epidermal cells expressing the plasmids indicated below. **A**. Box-GFP-UbΔGG is not internalized and localizes to the PM like Box-GFP (**B**) and does not localize to punctate structures like Box-GFP-Ub (**C**). **D-F**: Localization of the reporters given above *in planta*, confirming the results from (**A-C**). Vacuolar transport of RFP-TMD23-UbΔGG is not affected by the ΔGG mutation (**G**), compared to RFP-TMD23-Ub (**H**). Its phenotype thus differs from that of the PM marker RFP-TMD23 (**I**). **J-L**: Confirmation of the localization from **G-I** in leaf epidermal cells. Scale bars = 5 μm.

It is assumed that mono-ubiquitination mediates internalization of PM proteins, whereas poly-ubiquitination serves as signal for proteasomal degradation [10,51]. However, it has also been reported that PM proteins, with short polyubiquitin chains, are more efficiently internalized [19,52-54]. Moreover, deletion of the two C-terminal glycines of a recombinantly linked ubiquitin has been shown to prevent further ubiquitination, suggesting that these glycines act as an additional site for poly-ubiquitination [55]. Therefore, we have deleted both terminal glycine residues of Box-GFP-Ub (Box-GFP-UbΔGG) and RFP-TMD23-Ub (RFP-TMD23-UbΔGG). Interestingly, the expression of Box-GFP-UbΔGG in both tobacco protoplasts (Figure 4A) and leaves (Figure 4D) revealed a clear PM localization (Figure 4A and 4D) that was indistinguishable from the PM reporter Box-GFP (Figure 4B and 4E). Endosomal signals, as seen with the internalized Box-GFP-Ub construct (compare Figure 4A to 4C and 4D to 4F), were never observed. This suggests that the terminal glycine residues of ubiquitin are required for internalization of this molecule from the PM. The differential transport properties of Box-GFP-Ub and Box-GFP-UbΔGG furthermore demonstrate that ubiquitin acts as a specific sorting signal, rather than merely triggering a degradation response due to the individual properties of the reporter. In sharp contrast, deletion of the terminal glycine residues in RFP-TMD23-UbΔGG did not inhibit the ubiquitin-mediated internalization, as judged by the unperturbed vacuolar delivery of this reporter in protoplasts and *in planta* (Figure 4G and 4J; compare 4G to H and I and compare 4J to K and L).

### Ubiquitin directs Golgi-localized proteins to the vacuole

**Figure 5:**
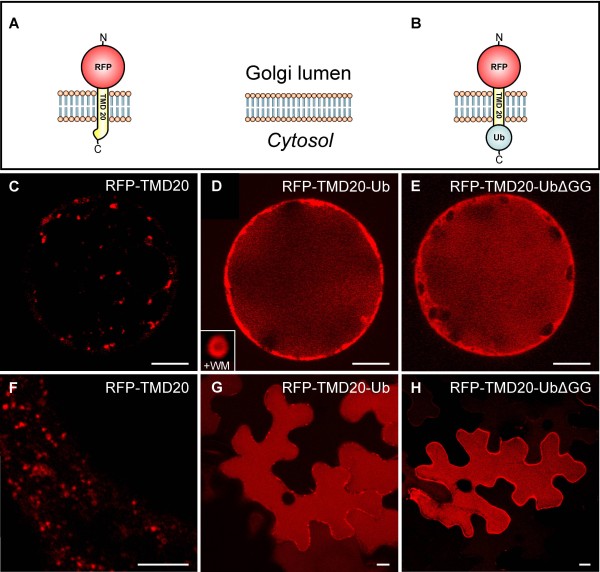
**Ubiquitin-dependent transport from the Golgi to the vacuole.A-B**: Schematic representation of the constructs RFP-TMD20 (**A**) and RFP-TMD20-Ub (**B**). Tobacco mesophyll protoplasts and leaf epidermal cells expressing the plasmids indicated below. RFP-TMD20 (**C**) exhibits a punctate pattern, while RFP-TMD20-Ub (**D**) is efficiently transported into the vacuolar lumen and also accumulates in ring-like structures after wortmannin-treatment (+WM; inset in **D**). Vacuolar transport of RFP-TMD20-UbΔGG (**E**) and RFP-TMD20-Ub is indistinguishable. **F-H**: Confirmation of the localization from **C-E** in leaf epidermal cells. Scale bars = 5 μm.

The previous results show that the fusion of ubiquitin to the cytosolic tail of PM reporters is sufficient for internalization, but the transport of the two ΔGG variants differs significantly. Therefore, it seemed plausible to assume that these differences are due to their different transport routes towards the PM. In contrast to the directly targeted Box-GFP-UbΔGG, the RFP-TMD23-UbΔGG transits the secretory pathway. We hypothesized that this reporter might already be sorted into the vacuolar transport route from a compartment *en route* to the PM. Therefore, we have used the Golgi marker RFP-TMD20 (TM20 in [48]) (Figure 5A) to generate the ubiquitin fusion protein RFP-TMD20-Ub (Figure 5B). Expression of RFP-TMD20 in tobacco protoplasts revealed a punctate pattern (Figure 5C), which showed colocalization with the Golgi marker Man1-GFP (Additional file 4). In contrast, expression of the ubiquitin fusion (RFP-TMD20-Ub) yielded a strong vacuolar signal (Figure 5D), but punctae, representing wortmannin-sensitive LEs/MVBs (inset in Figure 5D), could also be observed. Interestingly, the ubiquitin fusion construct lacking both C-terminal glycine residues (RFP-TMD20-UbΔGG) was equally well delivered to the vacuole (Figure 5E). The same localization patterns for RFP-TMD20, RFP-TMD20-Ub and RFP-TMD20-UbΔGG were observed *in planta* (Figure 5F-H), suggesting that ubiquitin-mediated vacuolar sorting exhibits differential demands on the C-terminus of ubiquitin, dependent on the location within the secretory pathway.

**Figure 6:**
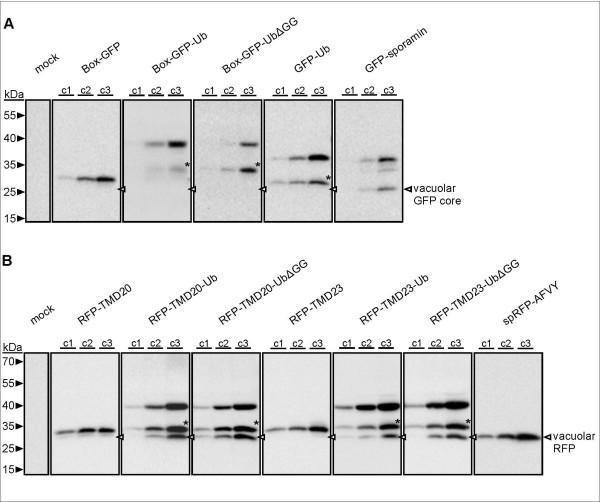
**Western-blot analysis to monitor for vacuolar delivery.** Tobacco mesophyll protoplasts were transfected with 10, 30 or 100 μg plasmid DNA encoding for each marker/reporter as indicated below or mock-transfected. Gel blot analysis using GFP (**A**) or RFP antibodies (**B**) reveals signals corresponding to the calculated molecular weight of the expressed proteins. Signals which might correspond to reporters with proteolytically cleavaged ubiquitin moiety are indicated by asterisks. Arrowheads indicate the size of the vacuolar GFP core and vacuolar RFP.

To analyze vacuolar delivery biochemically, we have compared the GFP-based reporters with the soluble vacuolar molecule GFP-sporamin (Figure 6A), which yields a characteristic vacuolar degradation product of GFP, termed GFP core [50]. Since vacuolar RFP does not yield a degradation product, we have compared all RFP-based reporters with the soluble vacuolar protein spRFP-AFVY, serving as a size marker for vacuolar RFP (Figure 6B). Protein gel blot analysis with GFP/RFP antibodies reveals specific signals of the calculated molecular weight for each of the reporters (Figure 6A and 6B). As concluded from the CLSM results (Figure 1, 2), neither Box-GFP-Ub, Box-GFP-UbΔGG nor the cytosolic GFP-Ub yields a signal in size of the GFP core, as indication for the lack of vacuolar arrival. All ubiquitin fusions show an additional signal, which is in each case about 8 kDa smaller than the calculated weight of the fusion protein (asterisks), approximating that of monomeric ubiquitin. In contrast to the GFP-fusions, all RFP-ubiquitin constructs show an additional third signal, which is precisely the size of the vacuolar reporter spRFP-AFVY. Together with the CLSM localization, the appearance of this lower molecular weight form indicates vacuolar arrival. This suggests that ubiquitin is capable to target proteins from the PM into the endocytic route but also targets proteins from the Golgi into the vacuolar transport pathway.

### Ubiquitin-mediated transport of membrane proteins to the vacuole occurs via the MVB

**Figure 7:**
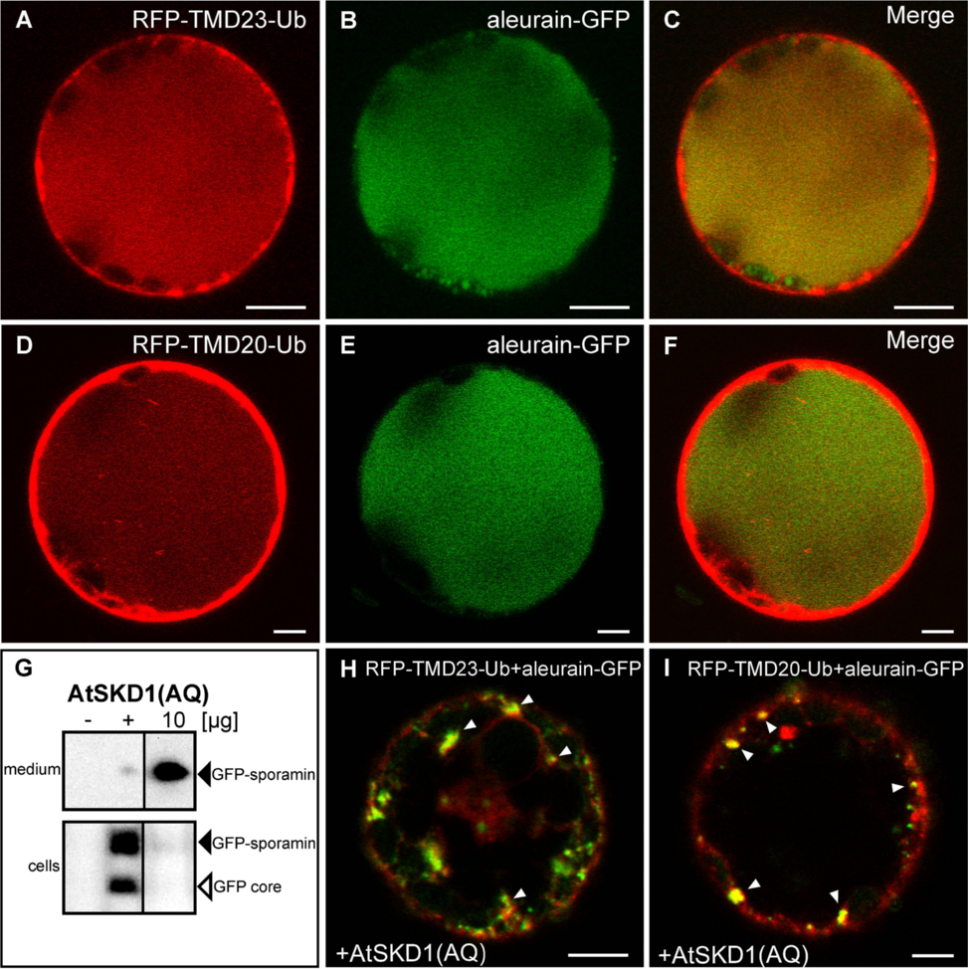
**Inhibition of ESCRT function inhibits vacuolar arrival of ubiquitin-modified reporters.** CLSM and western-blot analysis of tobacco mesophyll protoplasts expressing the indicated plasmids. **A-F**: RFP-TMD23-Ub (A-C, red) or RFP-TMD20-Ub (D-F, green). and the soluble vacuolar marker aleurain-GFP (green) colocalize in the vacuole. (**G**) The vacuolar GFP core of GFP-sporamin (open arrowhead) and the cellular transit form (closed arrowhead) disappear when coexpressed with 10 μg plasmid DNA encoding for the dominant-negative mutant AtSKD1(AQ). (−) mock-transfected, (+) positive control. **H-I**: AtSKD1(AQ) expression inhibits vacuolar delivery of RFP-TMD23-Ub (red) and aleurain-GFP (green) (H) or RFP-TMD20-Ub (red) and aleurain-GFP (green) (**I**). All reporters accumulate in punctae (arrowheads) under these conditions.

We wanted to test whether ubiquitin-facilitated transport from the PM and from the Golgi utilizes the ESCRT-dependent vacuolar route. It has been shown that expression of AtSKD1(AQ), a dominant-negative mutant of the ESCRT-III-associated AAA-ATPase, inhibits arrival of soluble vacuolar cargo [56]. In control experiments, co-expression of either of the vacuolar targeted RFP-TMD23-Ub and RFP-TMD20-Ub with the soluble vacuolar reporter aleurain-GFP [57] showed that both the membrane reporters and aleurain-GFP are delivered equally well to the vacuole (Figure 7A-C and Figure 7D-F, respectively). In the presence of AtSKD1(AQ), which inhibits vacuolar delivery of the soluble vacuolar protein GFP-sporamin and induces its secretion (Figure 7G), none of these reporters reached the vacuole but instead accumulated in intracellular compartments (Figure 7H and 7I).

## Discussion

### Targeting of membrane proteins in the secretory pathway

Secretion into the apoplast is regarded as being the “default pathway” for soluble proteins in the secretory pathway [58,59]. For plant membrane proteins, the situation is not that clear. It has been suggested that the tonoplast represents the default destination for this class of proteins [60,61] but this concept has recently been challenged. It was shown that mutants of the ER-resident p24 protein family that lack the di-lysine ER retrieval signal in their cytosolic domain and thus escape their first instance of sorting at the Golgi apparatus are indeed transported to the tonoplast but are also efficiently transported to the PM [62]. This dual localization of membrane proteins at the PM and the tonoplast is not restricted to mutants which have lost a specific sorting signal, since it can also be observed when fluorescent PM proteins like the plasma membrane ATPase (PMA) or the LOW-TEMPERATURE-INDUCIBLE PROTEIN (LTI6a) are analyzed [24]. The reasons for this differential localization are unclear as the sorting signals for these proteins have not yet been deciphered. It has also to be considered that all of these functional proteins are subject to cellular regulation mechanisms like quality control and turnover, which could contribute to vacuolar localization.

Although universal sorting signals for membrane proteins that allow for compartment-specific targeting are largely unknown, it has been demonstrated that the length of the TMD provides sufficient sorting information for targeting type-I proteins either to the ER, the Golgi or the PM [48]. This property has also been applied to the plant vacuolar sorting receptor (VSR) BP80, which localizes to the TGN/EE and the MVB/LE. In this case, various constructs carrying length-modified BP80-TMDs, but lacking the cytosolic tail, never deviated from this “default pathway”, suggesting that sorting into the vacuolar route requires additional information [48]. These examples furthermore show that the final location of a membrane protein is the result of a combination of sorting signals. For these reasons, we have decided to analyze the role of ubiquitin in protein targeting by the use of translational ubiquitin fusions, mimicking constitutive ubiquitination, based on reporters like Box-GFP or RFP-TMD20/23, which possess minimal but defined sorting signals for either the PM or the Golgi. The use of reporters that are *post*-translationally inserted into the PM (BOX-GFP) and reporters that are transported via the secretory pathway (RFP-TMD23/20) permits the exposure of identical sorting signals at different intracellular locations and thus allows the analysis of specific sorting signals for individual transport steps within the cell.

### Ubiquitin as a sorting signal for the endocytic pathway

We have addressed the question as to whether ubiquitin functions as a sorting signal for the endocytic pathway in plants by generating ubiquitin fusion proteins based on the PM-localized markers Box-GFP and RFP-TMD23. Despite differences in their transport towards the PM – Box-GFP is inserted *post*-translationally, whereas RFP-TMD23 transits via the secretory pathway – both molecules are internalized when fused to ubiquitin and colocalize with endosomal markers. This demonstrates that both reporters are indeed sorted into the endocytic transport route, which has been suggested to comprise the TGN/EE and the MVB/LE as transit compartments [5,37,63].

Even though both reporters undergo endocytosis, there are differences in their final location: RFP-TMD23-Ub yields fluorescent signals in the vacuolar lumen, while Box-GFP-Ub only reaches the MVB/LE and is never detected in the vacuole. One explanation for the failed vacuolar delivery of Box-GFP-Ub could be that this reporter is released from the MVB/LE membrane into the cytosol. This is possible, since the attachment of proteins to membranes via the Box sequence harbors two weak points: first, the reversibility of the membrane anchorage [64] and second, the specific interaction of the polybasic region with phospholipids of the membrane, which mediates the specificity during the recruitment [39,65]. In this regard, a gradual change in the lipid composition of the compartments along the endocytic route could trigger a release of the reporter from the membrane into the cytosol.

A translational ubiquitin fusion protein, which is *post*-translationally inserted into the PM via a lipid modification, has previously been used as a reporter to analyze internalization events in mammalian cells [55]. After expression, the reporter was found to be poly-ubiquitinated, while a derivative of the reporter that lacked the C-terminal glycine residues was not. However, both reporters appeared to be equally well internalized, indicating that a single ubiquitin moiety is sufficient to act as an endocytic sorting signal [55]. Inspired by this work, we have generated reporter derivatives lacking the C-terminal glycines of the ubiquitin. Box-GFP-UbΔGG localizes exclusively to the PM and is not internalized, compared to the Box-GFP-Ub. Therefore it is tempting to speculate that in plants, a single ubiquitin may not be sufficient to mediate sorting into the endocytic pathway. This view is also in agreement with recent findings based on translational ubiquitin fusions of the *Arabidopsis* plasma membrane ATPase (PMA-EGFP-UB), mutagenized in the ubiquitin moiety to prevent poly-ubiquitination [24]. The mutants, some of which were also lacking both C-terminal glycine residues, localized to the PM in addition to the vacuole in the majority of the cells, while a non-mutagenized ubiquitin fusion was mainly found in vacuoles and punctae but not at the PM. This suggests that the endocytic uptake of putatively mono-ubiquitinated reporters was also less efficient. This interpretation is also supported by the recent demonstration that the endocytic uptake and thus the stability of PIN2 depends on poly-ubiquitin chains [23] and the observation that IRT1 mutants, which lack two putative ubiquitination sides, fail to be internalized and accumulate at the PM instead [21]. This furthermore indicates that both multi-ubiquitination and poly-ubiquitination can act as internalization signals. Surprisingly, the transmembrane reporter RFP-TMD23-UbΔGG is still efficiently transported to the vacuole. At first glance, these observations are contradictory and difficult to reconcile, since both reporters carry identical sorting signals. However, in case of Box-GFP-UbΔGG, the signal is exclusively displayed at the PM, whereas in case of RFP-TMD23-UbΔGG, the signal is displayed throughout its journey towards the PM and might thus be captured and redirected before reaching the PM.

The finding that fusion of ubiquitin to a *post*-translationally inserted PM resident reporter is sufficient to trigger its internalization implies that plants possess an endogenous machinery capable of recognizing and sorting ubiquitin-tagged cargo. This hypothesis is supported by the recent identification of AvrPtoB, an effector of the plant pathogenic bacterium *Pseudomonas syringae* pv *tomato* DC3000, that acts as an E3 ubiquitin ligase [20]. The authors showed that AvrPtoB catalyzes poly-ubiquitination of FLS2. In combination with flg22, the effector induces the internalization of the receptor, leading to the suggestion that the degradation of FLS2 might be a mechanism of the pathogen to overcome plant innate immunity [20]. The demonstration that *Arabidopsis* lines lacking the cytosolic deubiquitinating enzyme AMSH3 are impaired in vacuolar biogenesis and, consequently, fail in vacuolar delivery of PIN2 [66] supports the significance of endogenous ubiquitin-mediated sorting processes.

### Ubiquitin as a vacuolar sorting signal

Taking into consideration that the deletion of one sorting signal redirects an ER-resident protein to the PM or an MVB/LE localized VSR to the Golgi, it is plausible to assume that the addition of a sorting signal is capable of overriding an existing one. The fusion of UbΔGG to the cytosolic tail of PM marker RFP-TMD23 represents just such an additional sorting signal, while it is the only existing sorting signal in the context of the *post*-translationally inserted PM reporter Box-GFP. Since the signal UbΔGG fails to drive internalization of Box-GFP-UbΔGG, it is plausible to assume that this also occurs in case of RFP-TMD23-UbΔGG. However, RFP-TMD23-UbΔGG was efficiently transported to the vacuole, which was also observed for the PMA-EGFP-UB mutants before [24], but it did not accumulate at the PM. We therefore speculated that a portion of the RFP-TMD23-UbΔGG molecules could have been sorted into the vacuolar pathway at a transit compartment prior to reaching the PM. It was recently suggested that direct trafficking from the Golgi to the vacuole does not significantly contribute to the vacuolar transport of ubiquitinated PM proteins, but it was also shown that a ubiquitin fusion of a MVB marker (AtVSR1-EGFP-UB) is also targeted to the vacuole [24]. If ubiquitin-dependent sorting of membrane proteins would be restricted to the PM, one would have to assume that ubiquitin firstly redirects AtVSR1-EGFP-UB from the vacuolar route towards the PM in order to trigger vacuolar delivery via the endocytic route. To test for ubiquitin-mediated sorting from the Golgi, we fused ubiquitin and UbΔGG to the cytosolic domain of the Golgi marker RFP-TMD20 [48]. This marker localizes to this compartment due to its TMD length of 20 amino acids, and does neither progress to the PM or into the vacuolar route. Both of the resulting reporters (RFP-TMD20-Ub and RFP-TMD20-UbΔGG) were efficiently sorted to the lytic vacuole and did not accumulate at the PM. These results show that ubiquitin-dependent vacuolar sorting can occur at the Golgi. We have shown that UbΔGG is insufficient to trigger the internalization at the PM. Since RFP-TMD20-UbΔGG does not accumulate at the PM, these results show that ubiquitin-dependent vacuolar sorting of this reporter does not occur via the PM.

The concept of ubiquitin-mediated sorting at the Golgi is also in agreement with our previous observation that the ESCRT-I subunit VPS28 localizes to the Golgi and the TGN/EE in *Arabidopsis* root cells, but that it is absent from MVBs/LEs, which can act as TGN-derived carriers that connect the TGN/EE in an clathrin-independent transport mode with the vacuole [28]. In yeast and mammals, ubiquitinated membrane proteins can already be recognized at the TGN by GGAs (Golgi-localized, γ-ear-containing ARF-binding proteins). These clathrin adaptors mediate protein sorting into clathrin-coated vesicles, which deliver their cargo to EEs where it is handed over to the ESCRT machinery [67,68]. The *Arabidopsis* genome, however, does not encode for GGA proteins [69]. In combination with a lack of homologous genes for ESCRT-0 and the ESCRT-I subunit Mvb12 [70], it is plausible to assume that the initial steps of ubiquitin-mediated sorting in plants differ from those in mammals and yeast.

The ubiquitin-dependent vacuolar delivery of the Golgi marker raised the question as to whether the transport of this chimera occurs through the biosynthetic vacuolar transport route via the TGN/EE and the MVB/LE. It was recently shown that a dominant-negative mutant of the ESCRT-associated AAA-ATPase SKD1 (AtSKD1(AQ)) is a potent inhibitor of transport of the soluble vacuolar reporter α-amylase-sporamin [56]. This soluble reporter is sorted into the vacuolar route via VSRs [50,71], but not via a direct interaction with the ESCRT machinery. However, this route collapses, if the ESCRT machinery is perturbed [56]. We have therefore applied this tool to analyze the transport route of the ubiquitin fusions RFP-TMD23-Ub and RFP-TMD20-Ub in direct comparison with the transport of the soluble vacuolar cargo aleurain-GFP. AtSKD1(AQ) prevented the vacuolar arrival of aleurain-GFP and both ubiquitin fusion proteins. This demonstrates that ubiquitin acts also as a sorting signal for the biosynthetic vacuolar route, when displayed at the surface of the Golgi/TGN.

This concept is supported by data obtained from yeast. There, newly synthesized membrane proteins are sorted in the TGN in a ubiquitin-based manner: proteins which are not ubiquitinated travel to the PM, whereas those which are, move down the endosomal pathway to the vacuole [72,73]. This is furthermore supported by the recent finding that the down-regulation of the PM-localized transporter BOR1 requires the combined action of tyrosine-based sorting signals as well as mono- and di-ubiquitination [22]. In this scenario, it is assumed that the tyrosine-based sorting signals confer internalization, while ubiquitin might be employed during endosomal sorting [22]. A similar role for ubiquitin has also been shown to operate in *Drosophila*[74]. It is therefore quite plausible that ubiquitin acts as a sorting signal already in the Golgi/TGN of plant cells.

## Conclusions

**Figure 8:**
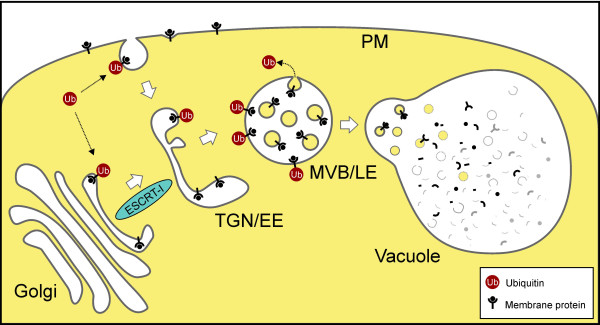
**Model illustrating ubiquitin-mediated vacuolar transport of membrane proteins.** Ubiquitin acts as an internalization signal at the PM for the endocytic route but also redirects Golgi-localized membrane proteins into the vacuolar degradation pathway. Both pathways merge at the TGN/EE. The ESCRT-I subunit VPS28 localizes to the Golgi and the TGN/EE but not to the MVB/LE [28]. This could suggest that ESCRT-mediated sorting is initiated at these compartments.

Our results show that ubiquitin acts as a sorting signal that mediates internalization at the PM but also redirects proteins from the early secretory pathway into the vacuolar degradation route (Figure 8). This might explain how ubiquitin as the sole sorting signal could lead to vacuolar delivery in a transport route that spans multiple compartments. Ubiquitin-mediated sorting at the Golgi/TGN might also hint to the existence of a ubiquitin-mediated, ESCRT-driven mechanism to enable the turnover of membrane proteins in the early secretory pathway, at a location beyond the ER-associated degradation (ERAD) pathway.

## Methods

### Plant materials

*Nicotiana tabacum* var. SR1 was grown under sterile conditions as previously described [75]. For leaf infiltrations, *Nicotiana benthamiana* was grown 5–6 weeks on soil. Wortmannin-treatment (30 μM) occurred for 1 h.

### Recombinant plasmid production

The following plasmids were used: Ara6-RFP [40], YFP-SYP61 [41], Man1-RFP and Man1-GFP [42], GFP-BP80 [50], aleurain-GFP [57], spRFP-AFVY and spL-RFP [49] and AtSKD1(AQ) [56].

Coding sequences were amplified by PCR from either first-strand cDNA [71] or plasmid DNA. Recipient vectors were cut according to restriction sites of fragments and dephosphorylated prior to ligation. All primers used are shown in Additional file 5: Table S1. The *Escherichia coli* strain MC1061 [76] was used for all plasmid amplifications. Box-GFP-Ub (pDS10) was assembled by ligating the Box coding sequence of AtROP10 [AT3G480409] - generated by annealed oligonucleotides -, the PCR-amplified GFP from pSN9 [32] and ubiquitin [AT5G03240] amplified from *Arabidopsis* cDNA into pAmy-HDEL [58]. Box-GFP-UbΔGG (pDS21) was amplified from pDS10 and cloned into pDS10 to replace Box-GFP-Ub. Box-GFP (pDS9) and cytosolic GFP-Ub (pFK17) were amplified from pDS10 and cloned into pAmy-HDEL and pPP11 ([71], respectively. RFP-TMD23 [77] and RFP-TMD20 (TM20 in [48]) were first subcloned into pPP11, resulting in pFK12 and pFK23. The coding sequence for ubiquitin and UbΔGG were amplified from pDS10 and ligated into pFK12 and pFK23, resulting in RFP-TMD23-Ub (pFK13), RFP-TMD20-Ub (pFK24), RFP-TMD23-UbΔGG (pFK19) and RFP-TMD20-UbΔGG (pFK25).

For leaf infiltrations, expression cassettes were subcloned into the binary Ti vector pGreenII 0029 [78]. This vector was previously modified to eliminate an intrinsic *Nhe*I site and to introduce MCS-flanking *Eco*RI and *Hind*III sites (pCN1). RFP-Hub1 (pFK26) was generated by PCR amplification of the C-terminal 1860 bps of CHC-1 [AT3G11130] from cDNA and ligation with the amplified RFP sequence from pFK13 into pGD5 [32].

### Transient gene expression, isolation of vacuoles, and leaf infiltration

Mesophyll protoplasts were isolated from 6–8 week-old plants and transfected via electroporation as described previously [77]. Unless otherwise stated, 10 μg of plasmid DNA were used for transfection followed by incubation for 24 h. Vacuoles were isolated 36 h after transfection as described previously [71]. Tobacco leafs were infiltrated with *Agrobacterium tumefaciens* (strain GV3101) as described previously [79].

### Protein extraction and immunoblot analysis

Cellular proteins were extracted in a final volume of 250 μL in 100 mM Tris pH 7.8, 200 mM NaCl, 1 mM EDTA, 2% (v/v) β-Mercaptoethanol and 0.2% (v/v) Triton X-100 by sonication. SDS-PAGE and immunoblot analysis were performed as described previously [75]. Antibodies were used as follows: anti-GFP (rabbit polyclonal [35]) 1:10,000 and anti-RFP (rat monoclonal, ChromoTek) 1:5,000. Peroxidase-conjugated antibodies against rabbit IgGs (Millipore) or rat (Sigma-Aldrich) were used according to the manufacturer. Signals were detected by the use of AceGlow (PEQLAB) in combination with the Chemocam imager (Intas).

### Confocal microscopy and immunofluorescence labeling

Imaging was performed using a Zeiss Axiovert LSM 510 meta CLSM as described previously [28]. *Post*-acquisition image processing was performed using the Zeiss LSM image browser and Corel-DrawX4. For the quantification of Box-GFP-Ub internalization, fluorescent punctate signals present in the cortical cytoplasm of n = 58 protoplasts were considered. Error bars were calculated as the standard deviation of the mean value and the p-value was computed using the *t-*test calculator from http://www.graphpad.com/.

### Statistical analysis of CLSM localization data

For statistical analysis, the PSC colocalization plug-in [80] for ImageJ [81] was used to calculate the linear Pearson correlation coefficient (rp) and the nonlinear Spearman’s rank correlation coefficient (rs) of red and green fluorescent signals. Values were between −1 (negative correlation) and +1 (positive correlation). The fluorescence values of all pixels across the two channels of all analyzed signals were depicted in a scatterplot. Masking of areas of was performed with the ImageJ brush tool as described by French et al. (2008). For every analyzed image, punctuate signals were selected and the threshold level, under which pixels were treated as background noise, was set to 10. At least 6 individual cells and a minimum of 100 signals were considered for every experiment.

## Competing interests

The authors declare that they have no competing interests.

## Authors’ contributions

DS, FK, CV, DR and PP designed and analyzed the experiments; DS, FK, CV and MSWY performed the experiments; SS and LJ contributed unpublished material; DS, FK DR, and PP and wrote the article. All authors approved the manuscript.

## Supplementary Material

Additional file 1 **Analysis of the Box-GFP-Ub expression pattern.** Tobacco mesophyll protoplasts were transfected with plasmids encoding for Box-GFP-Ub. The reporter was expressed for 24 h prior to CLSM analysis. Scale bars = 5 μm. Fluorescence signals of a tobacco protoplast are shown in a cortical view (A), in an optical section (B) and in a 3D projection (C), revealing localization of Box-GFP-Ub at the plasma membrane and in punctae. Click here for file

Additional file 2 **Quantitative analysis of the localization of Box-GFP-Ub and RFP-TMD23-Ub.** Tobacco mesophyll protoplasts were transfected with plasmids encoding for fluorescent markers/reporters as indicated below. Fluorescent proteins were expressed for 24 h prior to CLSM analysis. Scale bars = 5 μm. For quantification, the PSC coefficients (rp and rs) were calculated after analysis of at least 6 representative protoplasts and a minimum of 100 signals. The level of colocalization ranges from +1 for perfect correlation to −1 for negative correlation. The fluorescence values of all pixels across the two channels of all analyzed signals were depicted in a scatterplot. A: Box-GFP-Ub and the TGN-marker YFP-SYP61 show rp and rs values in a range that indicates colocalization. B: While the same is true for coexpression of Box-GFP-Ub and the MVB-marker Ara6-RFP, no positive correlation was observed with the Golgi marker Man1-RFP (C). Coexpression of RFP-TMD23-Ub with the same endosomal markers results in similar rp and rs values compared to A-B as predicted for an endocytic cargo molecule (D-E). Click here for file

Additional file 3 **Comparison of the ubiquitin-modified reporters and soluble vacuolar cargo.** Tobacco mesophyll protoplasts were transfected with plasmids encoding for fluorescent markers/reporters as indicated below. Fluorescent proteins were expressed for 24 h prior to CLSM analysis. Scale bars = 5 μm. Aleurain-GFP is delivered to the lumen of the vacuole (A), whereas Box-GFP-Ub reveals punctuate signals in the cortical cytoplasm (B). spL-RFP (the linker peptide from proricin fused to RFP, (C)) and RFP-TMD23-Ub (D) give the same expression pattern being localized to the vacuolar lumen. Click here for file

Additional file 4 **Analysis of Golgi markers and modified derivatives.** Tobacco mesophyll protoplasts were transfected with plasmids encoding for fluorescent markers/reporters as indicated below. Fluorescent proteins were expressed for 24 h prior to CLSM analysis. Scale bars = 5 μm. A-C: Coexpression of RFP-TMD20 with the Golgi marker Man1-GFP, demonstrating colocalization of both molecules. Click here for file

Additional file 5 **Table S1.** Primers used for cloning. Click here for file
