# Supplementary figures and images for: Ubiquitin initiates sorting of Golgi and plasma membrane proteins into the vacuolar degradation pathway
Source: BMC Plant Biol. 2012 Sep 12;12:164. doi: 10.1186/1471-2229-12-164 (PMC3534617; doi:10.1186/1471-2229-12-164)

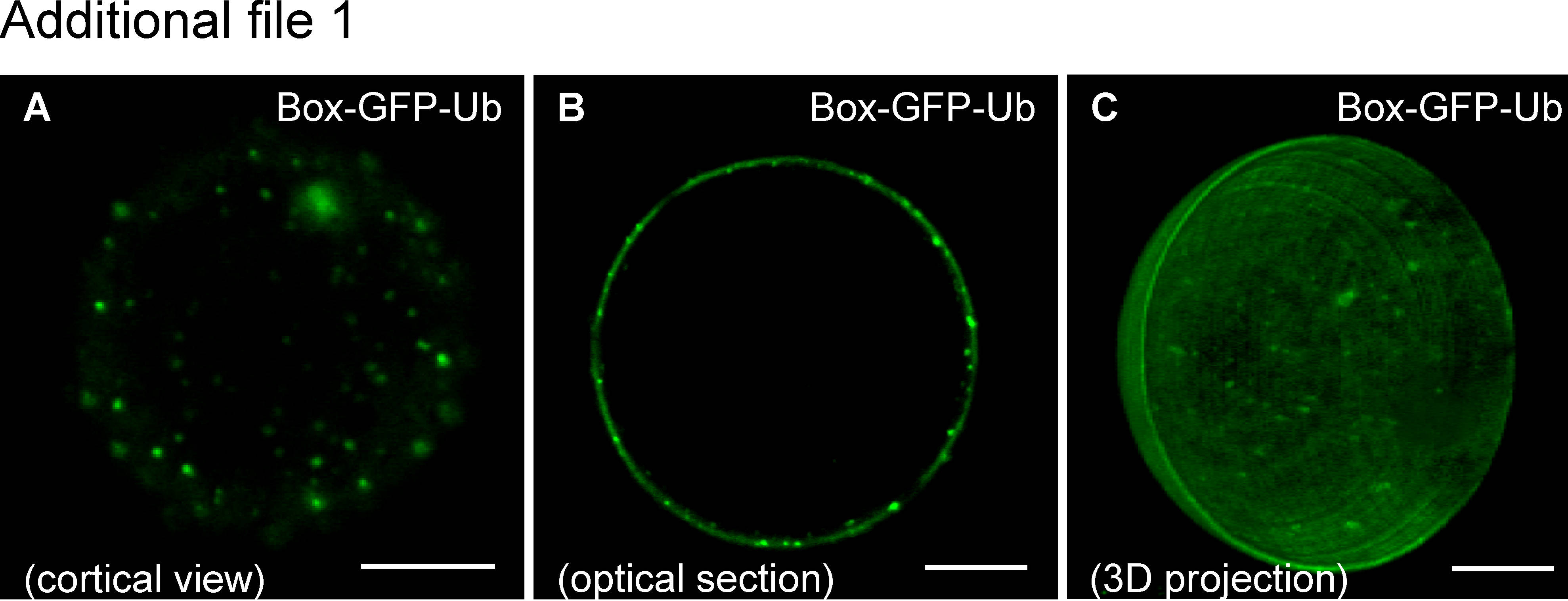

Supplement: Additional file 1 — Analysis of the Box-GFP-Ub expression pattern. Tobacco mesophyll protoplasts were transfected with plasmids encoding for Box-GFP-Ub. The reporter was expressed for 24 h prior to CLSM analysis. Scale bars = 5 μm. Fluorescence signals of a tobacco protoplast are shown in a cortical view (A), in an optical section (B) and in a 3D projection (C), revealing localization of Box-GFP-Ub at the plasma membrane and in punctae. [file 1471-2229-12-164-S1.jpeg]

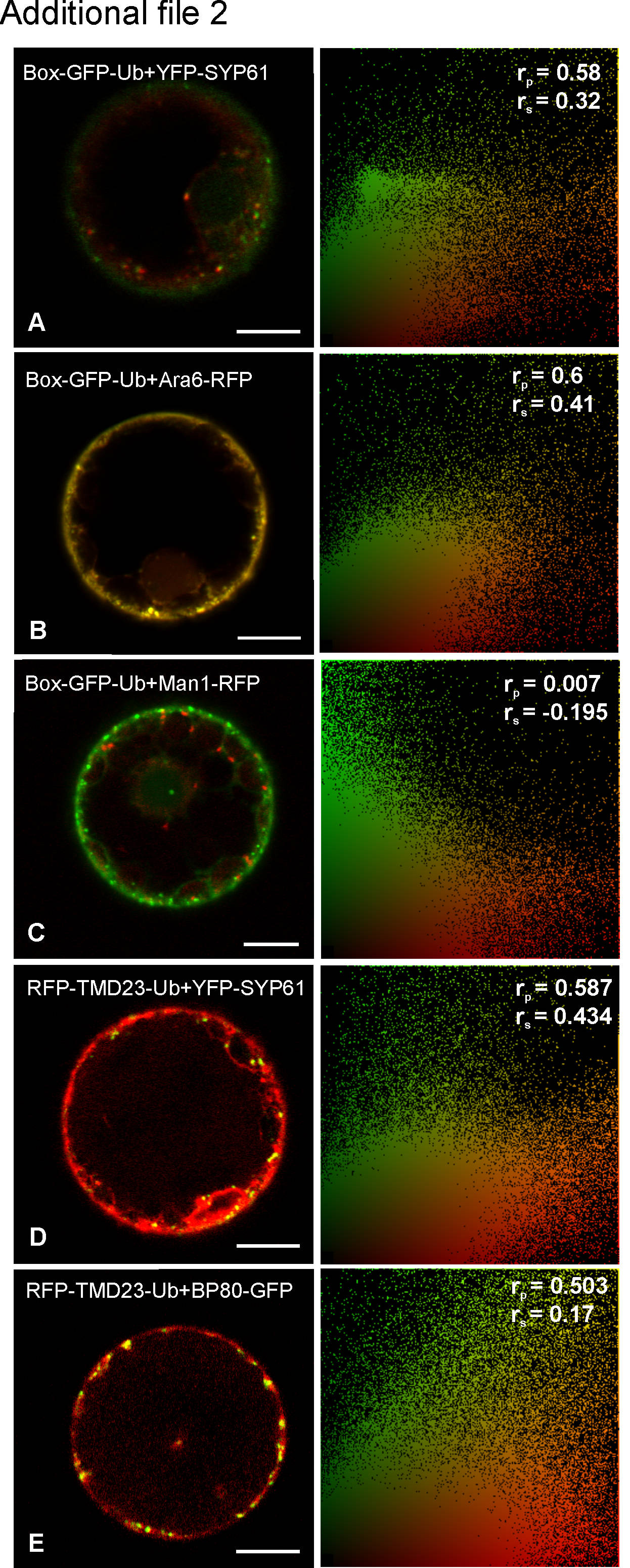

Supplement: Additional file 2 — Quantitative analysis of the localization of Box-GFP-Ub and RFP-TMD23-Ub. Tobacco mesophyll protoplasts were transfected with plasmids encoding for fluorescent markers/reporters as indicated below. Fluorescent proteins were expressed for 24 h prior to CLSM analysis. Scale bars = 5 μm. For quantification, the PSC coefficients (rp and rs) were calculated after analysis of at least 6 representative protoplasts and a minimum of 100 signals. The level of colocalization ranges from +1 for perfect correlation to −1 for negative correlation. The fluorescence values of all pixels across the two channels of all analyzed signals were depicted in a scatterplot. A: Box-GFP-Ub and the TGN-marker YFP-SYP61 show rp and rs values in a range that indicates colocalization. B: While the same is true for coexpression of Box-GFP-Ub and the MVB-marker Ara6-RFP, no positive correlation was observed with the Golgi marker Man1-RFP (C). Coexpression of RFP-TMD23-Ub with the same endosomal markers results in similar rp and rs values compared to A-B as predicted for an endocytic cargo molecule (D-E). [file 1471-2229-12-164-S2.jpeg]

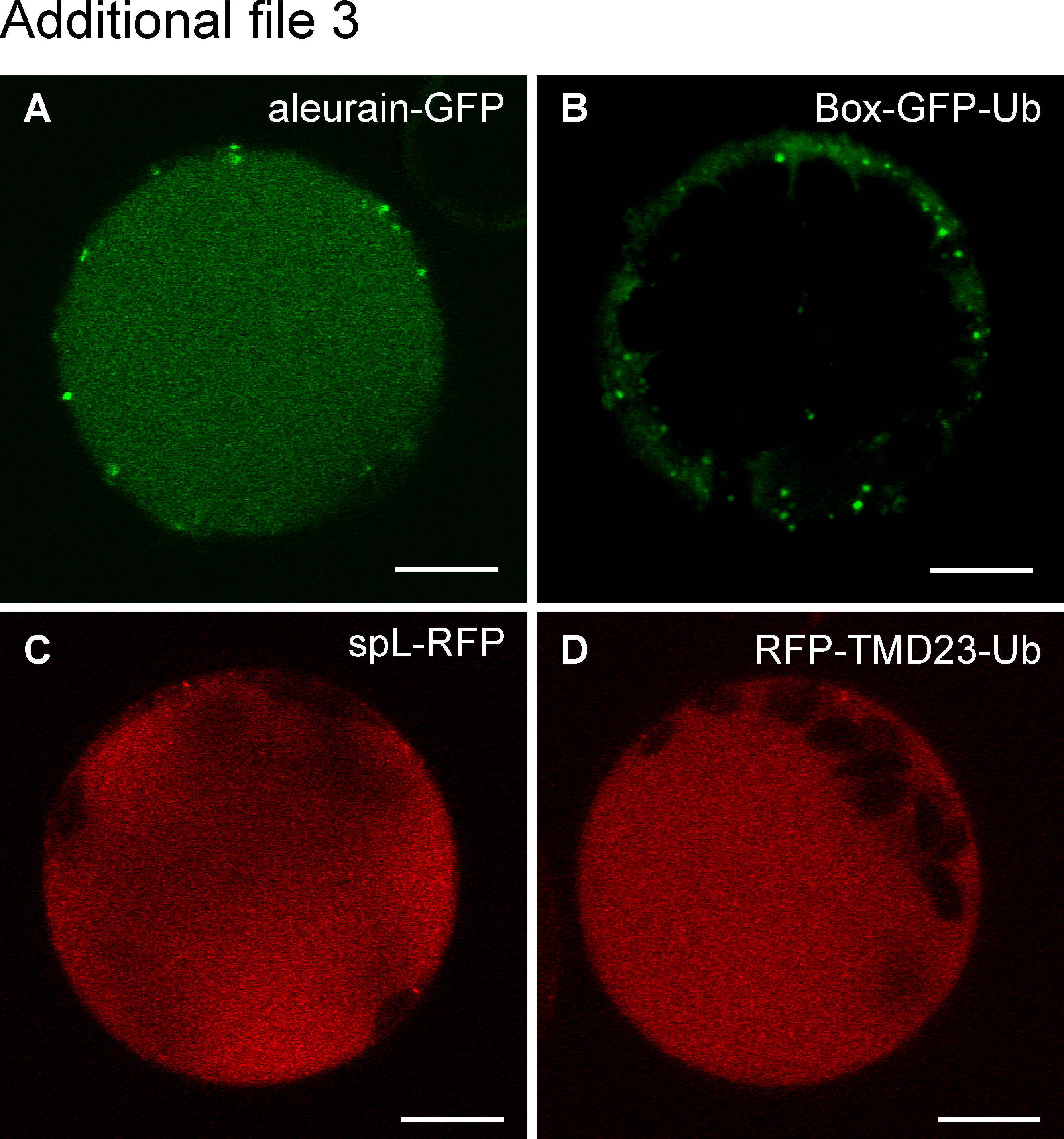

Supplement: Additional file 3 — Comparison of the ubiquitin-modified reporters and soluble vacuolar cargo. Tobacco mesophyll protoplasts were transfected with plasmids encoding for fluorescent markers/reporters as indicated below. Fluorescent proteins were expressed for 24 h prior to CLSM analysis. Scale bars = 5 μm. Aleurain-GFP is delivered to the lumen of the vacuole (A), whereas Box-GFP-Ub reveals punctuate signals in the cortical cytoplasm (B). spL-RFP (the linker peptide from proricin fused to RFP, (C)) and RFP-TMD23-Ub (D) give the same expression pattern being localized to the vacuolar lumen. [file 1471-2229-12-164-S3.jpeg]

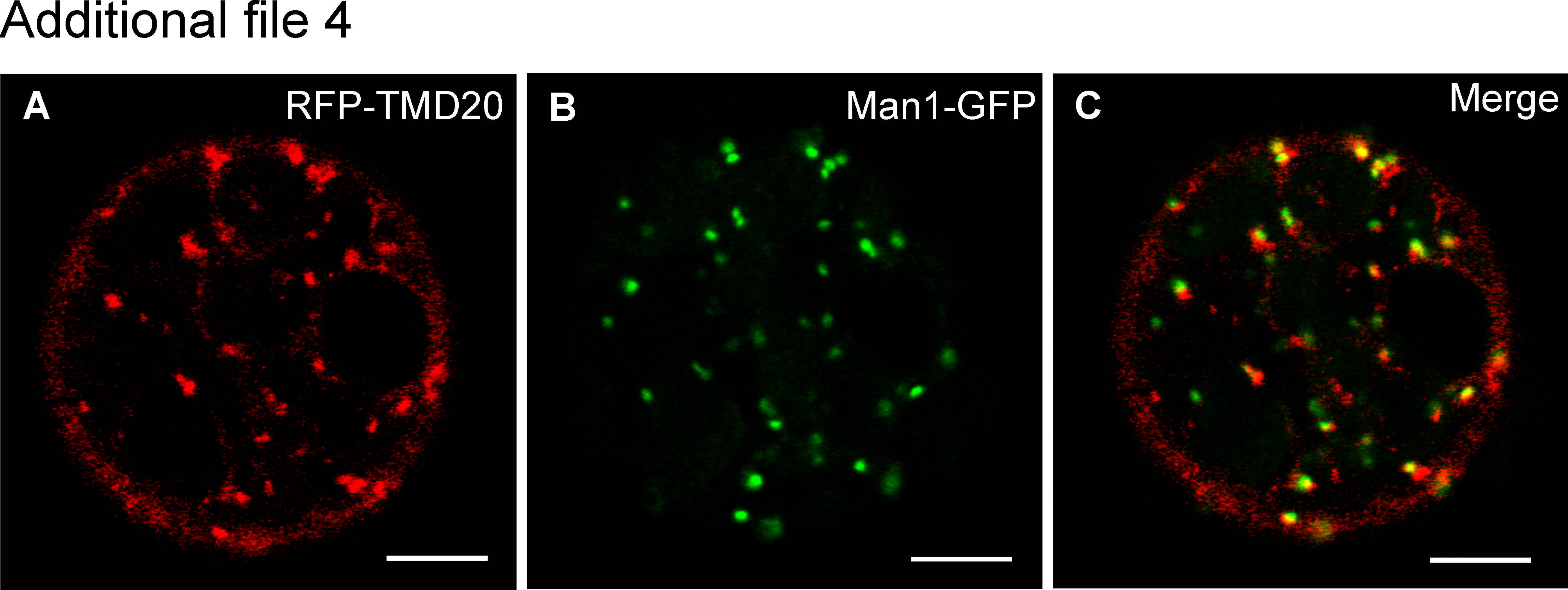

Supplement: Additional file 4 — Analysis of Golgi markers and modified derivatives. Tobacco mesophyll protoplasts were transfected with plasmids encoding for fluorescent markers/reporters as indicated below. Fluorescent proteins were expressed for 24 h prior to CLSM analysis. Scale bars = 5 μm. A-C: Coexpression of RFP-TMD20 with the Golgi marker Man1-GFP, demonstrating colocalization of both molecules. [file 1471-2229-12-164-S4.jpeg]
