# Supplementary material for: Ubiquitin initiates sorting of Golgi and plasma membrane proteins into the vacuolar degradation pathway
Source: BMC Plant Biol. 2012 Sep 12;12:164. doi: 10.1186/1471-2229-12-164 (PMC3534617; doi:10.1186/1471-2229-12-164)
Supplement: Additional file 5 — Table S1. Primers used for cloning. [file 1471-2229-12-164-S5.doc]

| **Oligonucleotide** | **Sequence (5’ – 3’ direction)** | **Template** | **Recipient vector** |
| --- | --- | --- | --- |
| **Box-GFP-Ub (pDS10):** | | | |
| Box_*Nco*I_*Cla*I_sense | CATGGCAGTGAAACAAAAGGAGAAGAAGAAGAAGCAGAAGCCTCGAAGCGGATGTCTCTCAAACATTCTGTGTGGGAAGAATGGAT | Complementary to oligo 2) | pAmy-HDEL |
| Box_*Nco*I_*Cla*I_antisense | cgatccattcttcccacacagaatgtttgagagacatccgcttcgaggcttctgcttcttcttcttctccttttgtttcactgc | Complementary to oligo 1) | pAmy-HDEL |
| GFP_*Cla*I_sense | TAGTGGATCGATGGTGAGCAAGGGCGAGGA | pSN9 | pAmy-HDEL |
| GFP_*Not*I_antisense | CCTATCGCGGCCGCCCTTGTACAGCTCGTCCATGC | pSN9 | pAmy-HDEL |
| Ub_*Not*I_sens | AGTCTAGCGGCCGCATGCAAATCTTCGTGAAAAC | cDNA | pAmy-HDEL |
| Ub_*Xba*I_antisense | CTAGTCTAGATTATCCACCACGAAGACGGA | cDNA | pAmy-HDEL |
| **Box-GFP-Ub∆GG (pDS21):** | | | |
| Box_*Nco*I_sense | CGAGCTCCATGGCAGTGAAACAAAAGG | pDS10 | pAmy-HDEL |
| Ub∆GG_*Bam*HI_antisense | CTCTAGTGGATCCTCAACGAAGACGGAGGACGAGAT | pDS10 | pAmy-HDEL |
| **Box-GFP (pDS9):** | | | |
| Box_*Nco*I_sense | CGAGCTCCATGGCAGTGAAACAAAAGG | pDS10 | pAmy-HDEL |
| GFP_*Bam*HI_antisense | TCGCGGGATCCTTACTTGTACAGCTC | pDS10 | pAmy-HDEL |
| **GFP-Ub (pFK17):** | | | |
| GFP_*Cla*I_sense | TAGTGCATCGATGGTGAGCAAGGGCGAGG | pDS10 | pPP11 |
| Ub_*Bam*HI_antisense | AGTCGCGGATCCTTAACCACCTCTTAAACGG | pDS10 | pPP11 |
| **RFP-TMD20 (pFK23):** | | | |
| SP_*Cla*I_sense | AGTCTAATCGATGAGGCTTTGTAAATTCACAGCTC | TM20 | pPP11 |
| TMD_*Bam*HI_antisense | AGTCTAGGATCCTTAAGATCTCTTCCTGCCGACGA | TM20 | pPP11 |
| **RFP-TMD20-Ub (pFK24):** | | | |
| Ub_*Bgl*II_sense | AGTCTGAGATCTATGCAAATCTTCGTGAAAAC | pDS10 | pFK23 |
| Ub_*Bam*HI_antisense | AGTCTAGGATCCTTATCCACCACGAAGACGGA | pDS10 | pFK23 |
| **RFP-TMD20-Ub∆GG (pFK25):** | | | |
| Ub_*Bgl*II_sense | AGTCTGAGATCTATGCAAATCTTCGTGAAAAC | pDS10 | pFK23 |
| Ub∆GG_*Bam*HI_antisense | AGTCTAGGATCCTCAACGAAGACGGAGGACGAGAT | pDS10 | pFK23 |
| **RFP-TMD23 (pFK12):** | | | |
| SP_*Cla*I_sense | AGTCTAATCGATGAGGCTTTGTAAATTCACAGCTC | TM23 | pPP11 |
| TMD_*Bam*HI_antisense | AGTCTAGGATCCTTAAGATCTCTTCCTGCCGACGA | TM23 | pPP11 |
| **RFP-TMD23-Ub (pFK13):** | | | |
| Ub_*Bgl*II_sense | AGTCTGAGATCTATGCAAATCTTCGTGAAAAC | pDS10 | pFK12 |
| Ub_*Bam*HI_antisense | AGTCTAGGATCCTTATCCACCACGAAGACGGA | pDS10 | pFK12 |
| **RFP-TMD23-Ub∆GG (pFK19):** | | | |
| Ub_*Bgl*II_sense | AGTCTGAGATCTATGCAAATCTTCGTGAAAAC | pDS10 | pFK12 |
| Ub∆GG_*Bam*HI_antisense | AGTCTAGGATCCTCAACGAAGACGGAGGACGAGAT | pDS10 | pFK12 |
| **RFP-Hub1 (pFK26):** | | | |
| mRFP_*Nhe*I_sense | AGTCTAGCTAGCATGGCCTCCTCCGAGGACG | pFK13 | pGD5 |
| mRFP_*Nco*I_antisense | AGTCTACCATGGCTCCAGTACTGTGGC | pFK13 | pGD5 |
| CHub_*Nco*I_sense | AAGCATCCATGGGATTCAAGAAGTTTAACTTAAA | cDNA | pGD5 |
| CHub_*Bam*HI_antisense | TTCCCAGGATCCTTAGTAGCCGCCCATCGGTG | cDNA | pGD5 |
| **Modifications in pGreenII, generating plasmid pCN1** | | | |
| **(1) Point mutation in the nos-Kan selection cassette to remove intrinsic *Nhe*I site:** | | | |
| nos_Prom_M_sense | AAGAAATATTTGCTATCTGATAGTGACCTTA | pGreenII 0029 |  |
| nos_Prom_M_antisense | TAAGGTCACTATCAGATAGCAAATATTTCTT | pGreenII 0029 |  |
| **(2) Deletion of the multiple cloning site:** | | | |
| pGIIBB_*Hind*III_sense | CGCCACAAGCTTGGAGCTCCAGCTTTTG | pGreenII 0029, modified in (1) | |
| pGIIBB_*Eco*RI_antisense | GGTACGGAATTCGCCCTATAGT | pGreenII 0029, modified in (1) | |
